# Competitive Exclusion among Fig Wasps Achieved via Entrainment of Host Plant Flowering Phenology

**DOI:** 10.1371/journal.pone.0097783

**Published:** 2014-05-21

**Authors:** Min Liu, Rui Zhao, Yan Chen, Jian Zhang, Stephen G. Compton, Xiao-Yong Chen

**Affiliations:** 1 School of Ecological and Environmental Sciences, Tiantong National Field Station for Forest Ecosystems, East China Normal University, Shanghai, China; 2 Ecological Security and Protection Key laboratory of Sichuan Province, Mianyang Normal University, Mianyang, Sichuan, China; 3 School of Biology, Faculty of Biological Sciences, University of Leeds, Leeds, United Kingdom; Key Laboratory of Tropical Forest Ecology, Xishuangbanna Tropical Botanical Garden, Chinese Academy of Sciences, China

## Abstract

Molecular techniques are revealing increasing numbers of morphologically similar but co-existing cryptic species, challenging the niche theory. To understand the co-existence mechanism, we studied phenologies of morphologically similar species of fig wasps that pollinate the creeping fig (*F. pumila*) in eastern China. We compared phenologies of fig wasp emergence and host flowering at sites where one or both pollinators were present. At the site where both pollinators were present, we used sticky traps to capture the emerged fig wasps and identified species identity using mitochondrial DNA COI gene. We also genotyped *F. pumila* individuals of the three sites using polymorphic microsatellites to detect whether the host populations were differentiated. Male *F. pumila* produced two major crops annually, with figs receptive in spring and summer. A small partial third crop of receptive figs occurred in the autumn, but few of the second crop figs matured at that time. Hence, few pollinators were available to enter third crop figs and they mostly aborted, resulting in two generations of pollinating wasps each year, plus a partial third generation. Receptive figs were produced on male plants in spring and summer, timed to coincide with the release of short-lived adult pollinators from the same individual plants. Most plants were pollinated by a single species. Plants pollinated by *Wiebesia* sp. 1 released wasps earlier than those pollinated by *Wiebesia* sp. 3, with little overlap. Plants occupied by different pollinators were not spatially separated, nor genetically distinct. Our findings show that these differences created mismatches with the flight periods of the other *Wiebesia* species, largely ‘reserving’ individual plants for the resident pollinator species. This pre-emptive competitive displacement may prevent long term co-existence of the two pollinators.

## Introduction

Plants and their insect pollinators display numerous examples of coevolution and coadaptation, most obviously in the diversity of floral structures and the various adaptations displayed by insects for obtaining rewards provided by the plants [1]. The phenology of flowering (the time of year when flowers are available to be pollinated) is also important if pollinators and plants are to interact successfully [2]–[4], especially for those plants that rely on just one or a small number of insect species for pollination [5]. In the case of nursery pollination systems, where the reward for the insects is a place to breed, any mismatch in timing has serious consequences for both partners in the mutualism [6].

Fig trees (*Ficus* spp., Moraceae) and their host specific fig wasp pollinators (Hymenoptera, Agaonidae) have a history of joint dependency dating back more than 60 Myr [7]. Largely tropical and sub-tropical in distribution, they are perhaps the most extensively studied of all obligate mutualists [8]–[10]. For many years it was thought that each species of fig tree was pollinated by a single unique species of fig wasp that was associated with no other trees [8]. Early documented exceptions were assumed to result from fig wasps mistakenly entering figs of atypical hosts [11], or from mismatches in the formal taxonomy of fig trees and pollinators (with what were considered to be *Ficus* ‘species’ having different pollinators in different parts of their range). Subsequently, well-documented examples of fig species with more than one morphologically-distinct pollinators have accumulated, with up to four species of fig wasps recorded pollinating a single crop of figs [12], and some *Ficus* species found to have different pollinators depending on the habitat where they are growing [13], [14]. More recently, molecular markers had revealed the presence of many genetically divergent fig wasp species sharing the same host figs [15], [16]. Morphological differences between these species are small or absent and they are often referred to as ‘cryptic’ species of pollinating wasps [15], [17]. The findings of several cryptic pollinating wasps in a fig tree create a paradox for the ecological competition theory, i.e., coexistence of seemingly identical competitors [18]. However, whether those cryptic fig-wasps can coexist has been rarely checked (but see [19]). We suggest that those cryptic pollinating wasps may differentiate in some manners, if the coexistence of cryptic fig wasps is stable.

Fig trees rely on adult female fig wasps for dispersal of their pollen, and in turn the pollinating wasps can only lay their eggs in the florets located within the figs. Adult female pollinating wasps have an extremely short life span that typically lasts less than 48 hours [20], but see [21]. Persistence of the mutualism is therefore dependent on a close match between the flowering phenologies of fig trees and their pollinators, in combination with the fig wasps’ extremely effective dispersal and host-finding abilities [22]. As a result, changes in fig tree phenology due to abnormal climatic conditions can lead to drastic fluctuations in fig wasp populations and even local extinctions [23].

Individual monoecious fig trees (with figs that contain both seeds and fig wasp progeny) typically produce synchronized fig crops, each of which has a relatively brief period when they are attractive to wasps (receptive) and another brief period when they are releasing the next generation of wasps to disperse. Population persistence is achieved by different trees fruiting at different times, so that fig wasps emerging from one tree have a chance of finding receptive figs on another tree elsewhere [24]. Flowering phenologies are more diverse among dioecious fig tree species, where there are distinct female and male trees that specialize in seed or fig wasp (plus pollen) production respectively. Depending on the species, fig production may or may not be synchronized within individual trees, and the timing of flowering may differ between the sexes [25], [26].

Using mtDNA COI and nDNA ITS sequences, Chen *et al*. [27] showed that three genetically distinct but morphologically similar sister species of fig wasps pollinate *F*. *pumila* in southeastern China. These cryptic species are referred to as *Wiebesia* spp. 1, 2 and 3. The distributions of *Wiebesia* spp. 1 and 2 overlap slightly, as do the distributions of *Wiebesia* spp. 1 and 3, but areas of sympatry are narrow compared with their overall ranges, resulting in most populations of *F. pumila* being pollinated by a single species of fig wasp [27]. The current distributions of the three *Wiebesia* species may reflect post-glacial range expansions following the survival of their host plant in three isolated glacial refugia, each of which maintained a single species of pollinator. Genetic evidence suggests that after the climate warmed, *Wiebesia* spp. 1 and 2 rapidly extended their ranges as their host plant expanded northwards, but *Wiebesia* sp. 3 has declined in abundance during this period [27], [28].

It is unlikely that the current narrow areas of overlap between the three pollinator species results from the three species only recently coming into sympatry, following expansion by *F. pumila* from its refugia, because the rapid post-glacial vegetation changes documented for southern China [29] suggest that *F. pumila’*s current range is long-established. Alternative (and not mutually exclusive) explanations include competitive exclusion of one pollinator by another away from contact zones, differences in the physiological tolerances of the wasps, restricting them to different climate zones within their host’s overall range, and differences in suitability for particular fig wasps among host plant populations. The latter includes possible mismatches between the flight periods of the fig wasps and the flowering phenology of their host plants, which is particularly important in *F. pumila,* because it produces receptive figs for only brief periods each year.

In this paper, we study the emergence phenologies of sympatric fig-wasp species associated with *F. pumila* to check whether they differentiate. Molecular identification methods of cryptic species of fig wasps have created the opportunity to evaluate the extent to which these species co-occur and how these species may coexist. Here we used molecular markers to distinguish between *Wiebesia* spp. 1 and 3 in a region where these two *F. pumila* pollinators are both present. This allowed us to compare the flight periods of the two fig wasps and to assess whether the differences we observed may contribute to their reproductive isolation, modify inter-specific competition between them and broaden the period when their host plant can be pollinated.

## Materials and Methods

### Ethical Statement

*Ficus pumila* and its pollinating wasps are not protected. The study sites were neither privately-owned nor protected area. Therefore, no specific permissions were required for these locations/activities.

### Study Species

*Ficus pumila,* known as the creeping fig, is a dioecious fig tree native to eastern Asia. Our study sites in Eastern China have been subject to extensive human disturbance and *F. pumila* is found mainly in secondary vegetation, on agricultural land and on buildings and rocks. As its common name suggests, it forms dense mats up to about 20 cm high and tens of square meters in area that cover walls, rocks and trees. The figs of *F. pumila* are produced in the leaf axils. They are large, reaching about 50–70 mm in diameter at maturity. Crop sizes vary from just one or two figs up to several thousands on the largest plants.

Like other dioecious fig trees, male *F. pumila* produce figs that support the development of pollinator fig wasp larvae and produce no seeds. The *Wiebesia* species associated with *F. pumila* are passive pollinators. After completing their development, emerging adult female fig wasps become covered with pollen from the numerous male flowers in the figs, and then emerge in search of receptive figs. The reproductive success of the male plants is dependent on dispersal of the wasps to female trees, where they can enter receptive female figs and pollinate them. The female fig wasps are unable to oviposit in these figs, so they only produce seeds, and the wasps cannot opt to disperse a second time, because they lose their wings when they enter into the figs. Pollinator females that enter receptive figs on their natal trees provide no immediate reproductive award for male plants, but nonetheless help to maintain a resident population of fig wasps whose progeny can potentially contribute to pollination of female plants in the future [26].

Female *F. pumila* produce a single annual crop in spring, which will mature in autumn, but the flowering phenology of male *F. pumila* has been reported to vary with latitude, with spring and summer generations of fig wasps said to be produced each year in the south, but only one generation in more northerly areas [30], [31]. Male figs can contain more than 3000 female flowers and ten or more foundress female pollinators often enter each receptive fig to oviposit. The number of offspring they produce varies from about 100 to more than 2000 (X-W. Le et al., unpublished data).

*Wiebesia pumilae* was first described and recorded as the pollinator of *F. pumila* by Hill (1967) from Hong Kong, naming it *Blastophaga pumilae*. *Wiebesia* sp. 2 is probably the true *W. pumilae* as it is the only one of the three Chinese species associated with *F. pumila* that is known from Hong Kong [27]. It occurs in southern mainland China and is replaced further north in Jiangxi, Anhui, Jiangsu and Zhejiang Provinces by *Wiebesia* sp. 1. *Wiebesia* sp. 3 has a more limited distribution and is found mainly on offshore islands of the Zhoushan Archipelago in Zhejiang Province [27]. Within the archipelago, some islands support both species, but *Wiebesia* sp. 1 is more widespread than *Wiebesia* sp. 3, which is restricted mainly to the more eastern and isolated islands [32]. No non-pollinating fig wasps were reared from *F. pumila* figs in Zhejiang Province during our study.

### Flowering Phenology

**Figure 1:**
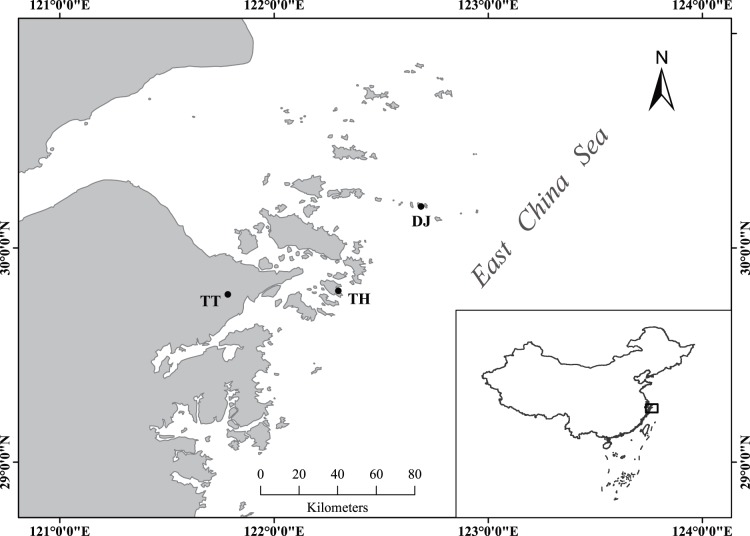
Locations of the study sites, Tiantong, Taohua and Dongji, on the Pacific coast of China, south of the city of Shanghai.

The flowering phenology of *F. pumila* was monitored at three coastal sites in Zhejiang Province, south of Shanghai, in 2009 (Fig. 1). Tiantong (N29.78°, E121.78°) is a mainland site where only *Wiebesia* sp. 1 has been detected since recording began in 2006. Dongji (N30.19°, E122.69°) is an offshore island where only *Wiebesia* sp. 3 is present and Taohua (N29.80°, E122.30°) is an island that supports populations of both species. The two most widely-separated sites are about 100 km apart. The consistency in distribution of these pollinator species over several years of recording suggests that their dispersal abilities are limited relative to those of some other fig wasps, a conclusion that is supported by genetic evidence [33].

We monitored the development of the figs on 14 male trees at Tiantong, 10 on Taohua Island and 10 on Dongji Island. The four major phases of development [34] were determined by external examination of the figs. A) Pre-receptive phase figs were small and green, with tightly closed ostiolar bracts. B) Receptive phase figs, when foundress females enter the figs to lay their eggs, were also green but larger and softer, with a distinctly flattened ostiolar region and a distinctive odor. C) Inter-floral phase figs, that contained developing fig wasp larvae inside galled ovules, were larger and harder. D) Male phase figs were releasing wasps. After releasing wasps, the figs dropped from the branches and rotted. Twenty C phase overwintering figs were labeled on each tree in December 2008 and up to 20 pre-receptive figs of the next generation were labeled in early spring 2009 (the spring crop of figs on male trees is small and sometimes entirely absent). From April to late May and July to late August we then recorded the development of the figs every four days, with daily recording whenever B and D phase figs were present. A small number of figs aborted. They were replaced by adjacent figs of similar size and development. During other time periods we visited the trees once a month to record their development. At the same time, five female trees in each place were chosen to monitor their phenology. In addition, the sizes of spring (May) and summer (July to August) crops of receptive figs were recorded on 45 male and 23 female trees in 2011 on Taohua Island.

### Flight Periods and Emergence Dates

The spring flight periods of adult fig wasps were monitored on Taohua Island, where *F. pumila* is pollinated by both *Wiebesia* spp. 1 and 3. In spring 2009, two or three sticky traps (depending on plant size) were placed among the branches of ten male trees, chosen because they had at least 50 overwintered and accessible figs. The sticky traps were translucent plastic sheets (30×44 cm) coated with an odorless glue (Shengshanchun Company, Cixi City, Zhejiang, China). They were replaced daily from April 20th (before the first fig wasps began to emerge) until May 28th, when all of the older generation of figs had dropped from the plants. The timing and location of the sticky traps meant that most of the wasps that were trapped had emerged from male phase figs on the same trees, but some may have emerged from figs on other trees and had arrived in search of receptive figs to enter. After removal from the traps, the fig wasps were stored in absolute ethanol at 4°C prior to identification.

The wasp communities in over-wintered individual figs on eight male trees were monitored in spring 2011 (using five of the trees monitored with sticky traps in 2009, plus three additional trees). Fifteen C phase figs on each tree were enclosed inside gauze bags to prevent any emerging wasps from escaping and were then checked daily. When the wasps emerged, they were collected and stored in absolute ethanol for further identification. A maximum of two figs per tree per day were collected and any additional figs with emerging wasps had their bags removed to allow them to escape. In total, between five and eight figs were sampled from each tree.

Because visual methods cannot reliably distinguish the two cryptic species of pollinating wasps, we identified individuals using the mitochondrial gene cytochrome oxidase I (COI), which has previously been shown to be effective in separating these species [27]. Total DNA was extracted from whole bodies of the fig wasps using a modified method of Sambrook *et al*. [35]. For wasps collected in 2009, part of the mitochondrial COI gene, which showed an average Kimura-2-parameter (K2P) distance of 11.6% between *Wiebesia* sp. 1 and *Wiebesia* sp. 3 [27], was amplified using the primer pair C1-J2183 (Jerry) and TL2-N-3014 (Pat) [36]. The PCR reactions were carried out in a 50 µL volume with approximately 60 ng of genomic DNA as the template, containing 0.25 mM each dNTP, 0.6 µM each primer, 1× PCR buffer, 2 mM MgCl_2_, 2 U of DNA Taq polymerase (Sangon, Shanghai, China). The reactions included an initial denaturation of 3 min at 94°C; 30 cycles of 30 s at 94°C, 45 s at 57°C, 1 min at 72°C; and a final extension of 72°C for 4 min. PCR products were sequenced on an ABI 3730 DNA Sequence Analyzer (Applied Biosystems, Foster City, CA). Sequences were manually compiled and aligned using CLUSTAL X v.2.0. Analyses were limited to reliably aligned regions from the data set and regions that could not be unambiguously aligned were excluded from the analysis. The K2P distance was calculated using MEGA v.4.0 [37].

We established the identity of fig wasps collected in 2011 using the microsatellite *WP294* [38], whose products had a difference in length of at least 16 bp between *Wiebesia* sp. 1 (polymorphic, with allele size range: 129–157 bp) and *Wiebesia* sp. 3 (monomorphic with allele size of 113 bp), via agarose gel electrophoresis. 23 wasps were identified using both microsatellite locus *WP294* and mtDNA COI: consistent identifications were obtained (M. Liu *et al*. unpublished data).

### Genetic Differentiation in Populations of the Host Plant, *Ficus pumila*

Twenty-five haplotypes of the cpDNA sequence based on three fragments, trnS-trnG, atpH-atpF and trncF-ycf6R, have been recorded in *Ficus pumila* populations in China, but individuals in the northeast part of its distribution in China, including Tiantong, Dongji and Taohua populations, all share the same haplotype (F.E. Peng, unpublished data). In addition, nuclear microsatellites assignment test using STRUCTURE inferred two clusters, east and west, with Daiyun and Tianmu mountains as boundary (J. Zhang, unpublished data), and the three populations were all in the east cluster, indicating low genetic differentiation. We further explored the genetic variation between *F. pumila* individuals by evaluating eleven microsatellite variation [39] on 25, 32 and 25 plants respectively from the three populations (Table S1in File S1).

We used FSTAT v2.9.3 [40] to test for linkage disequilibrium of the eleven loci. We compensated for multiple testing by using the false discovery rate (FDR) procedure implemented in the *R* package QVALUE [41]. MICRO-CHECKER [42] was used to detect null alleles. We carried out a genetic-distance based principal coordinate analysis (PCoA) to examine the clustering pattern of the three populations and pairwise genetic differences between individuals were calculated in GenAlEx v 6.41 [43].

## Results

### Flowering Phenology and Fig Wasp Flight Periods

**Figure 2:**
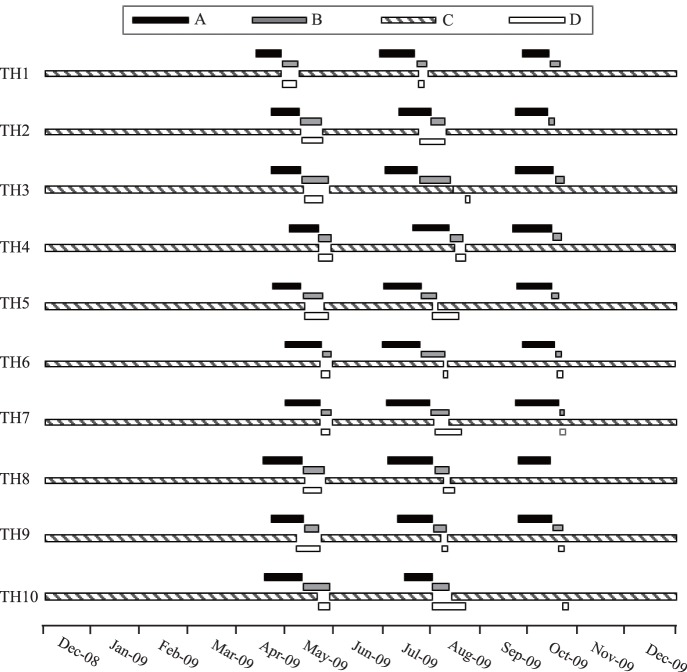
The fruiting phenologies of 10*F. pumila* individuals at Taohua. A, B, C and D indicate the phases of fig development, with B phase figs receptive to pollinators and D phase figs releasing pollinators.

Flowering on male trees was broadly synchronized at both the individual tree and population levels, but fig generations overlapped to an extent that allowed pollinator populations to cycle on individual trees. Contrary to previous reports, male *F. pumila* produced two major crops annually, with figs receptive in spring and summer. A small partial third crop of receptive figs occurred in the autumn, but few of the second crop figs matured at that time. Hence, few pollinators were available to enter third crop figs and they mostly aborted (Fig. 2, Figs. S1, S2 in File S1). This flowering pattern resulted in two major generations of pollinating wasps each year, plus a partial third generation.

Most female trees produced only one crop per year, resulting from figs that were receptive in spring and matured in the autumn. Hence, only the pollinating wasps that had overwintered as larvae and then emerged from male figs in the spring had the potential to contribute to the reproductive success of their host plants. The second (and partial third) generations of pollinators that emerged in the summer (and autumn) were only of value to their natal plants if they oviposited into figs on the same plants, because they could then contribute offspring to the spring generation. Only the spring crop of male figs contained male flowers and consequently only female wasps of that generation carried pollen when they emerged from the figs. This meant that the very small number of receptive figs that were produced on some female trees in the summer were unable to set seed, because the pollen was immature at that time and any fig wasps that entered them were not carrying pollen. These figs all aborted.

Crop sizes differed between sexes and seasons. In spring 2011 the number of receptive figs on female plants in spring (150.2±31.3 figs, mean ± SE, n = 23 crops) was about ten times more than that on male plants (15.4±5.2 figs, n = 45 crops). Some male trees even produced no receptive figs at all in spring (Fig. S3 in File S1). These contrasting fruiting patterns ensured that many of the fig wasps that were released in spring needed to disperse elsewhere to find receptive figs, thereby increasing the likelihood of these fig wasps entering figs on a female plant. Those fig wasps that did enter figs on male trees, and managed to reproduce, produced offspring that developed rapidly through the warm spring and early summer, resulting in a summer generation being produced after a relatively short generation time of about three months. The adult fig wasps that emerged in summer had much larger crops of receptive figs available to them on their natal male trees (201.4±34.0 figs, n = 45 crops), and rare female figs to trap them. This allowed the fig wasp populations to expand and recover from the mortality generated by the host plant’s spring fruiting patterns. It was mostly fig wasps of this summer generation that overwintered, with larvae staying in the figs for periods of up to nine months, though some emerged in autumn and could then produce a partial (autumn) third generation if they managed to enter one of the small number of young receptive figs that were produced at that time. The contrasting seasonal fig production of the sexes is emphasised by the relative proportions of figs they produced: the spring generation contributed 7.12% of the total number of figs recorded on male plants, compared with 98.18% on female plants (n = 9756 and 3519 figs, respectively).

**Figure 3:**
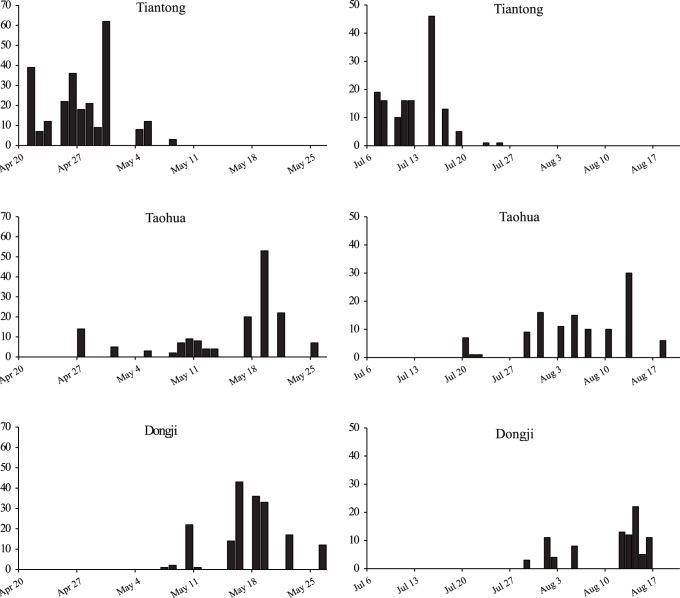
The timing of emergence of pollinators from figs of *Ficus pumila* in spring and summer in 2009: Tiantong (14 trees), Taohua (10 trees) and Dongji (10 trees).

The dates when fig wasps emerged from the figs in spring 2009 varied among the three populations (Fig. 3). In Tiantong, wasp emergence took place from April 21th to May 8th (n = 249 figs). Emergence tended to be later in the Dongji population, where wasps emerged between May 7th and May 26 th (n = 181 figs). The Taohua population had a longer spring emergence period, from April 27th to May 26th (n = 158 figs), that spanned the periods recorded from the other two populations (Fig. 3, left panel). This contrast in temporal patterns between sites was repeated by the summer generation of fig wasps, with pollinator emergence from the Tiantong population again taking place before that from Dongji, and with an extended period of emergence on Taohua (Fig. 3, right panel).

The flowering periods of female trees also differed among the three populations, and matched with the phenology of male trees. In 2009, in population Tiantong, new fig began to grow on March 24th and entered the receptive phase in the middle of April. In population Taohua, new figs began to grow from March 26th to middle April and entered the receptive phase in middle May. In population Dongji, new figs began to grow about late April and entered the receptive phase in late May. In Taohua, the flowering period of female trees at the study site appeared to miss the early male trees, but in 2011, when we investigated crop size over a broad range, we actually found some early female trees. Thus, both the early emergence wasps and the late wasps had the potential to be effective pollinators. After being pollinated, figs began to develop and matured in September to October.

**Figure 4:**
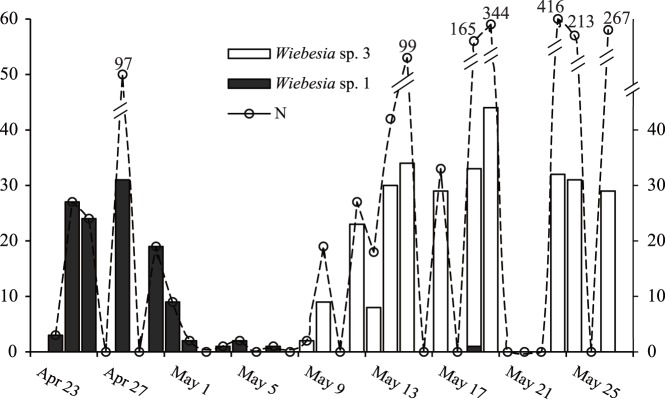
The numbers and identity of female pollinator fig wasps captured on sticky traps placed in male *F. pumila* trees in spring 2009 on Taohua. The bars record the sub-sampled individuals that were identified by mtDNA COI, and the dashed line and associated numbers indicate the total numbers of wasps trapped at each date.

A total of 1830 pollinating wasps were collected on Taohua Island during the 22 days of the 2009 spring emergence period when the sticky traps were in place on the plants. The COI genes of 423 individuals, sub-sampled from the fig wasps collected each day, were sequenced to identify the species present. We found a total of 10 haplotypes (Genbank accession number HQ398108-HQ398117). Pairwise divergences of within-clade haplotypes varied from 0.1% to 1.4%, and those of between-clade haplotypes varied by 11.8% to 12.6%. These corresponded with Chen *et al*.’s (2012) assignments of haplotypes H1 to H4 to *Wiebesia* sp. 1, and haplotypes H5 to H10 to *Wiebesia* sp. 3. Subsamples (n = 119 wasps) from the 185 fig wasps recorded on the sticky traps before May 7th all belonged to *Wiebesia* sp. 1, whereas subsamples (n = 304 wasps) from the 1645 individuals trapped at later dates almost all belonged to *Wiebesia* sp. 3 (Fig. 4). The exception was a single late individual of *Wiebesia* sp. 1, trapped on May 19th.

Spring adult female emergence dates at Taohua in 2011 were slightly later than in 2009, but displayed the same pattern (Fig. S5). Emergence was recorded from a total of 49 figs. *Wiebesia* sp. 1 females emerged from 19 figs on 6 trees, and *Wiebesia* sp. 3 from 31 figs on 7 trees. Five trees were shared by the two wasp species, but in total only one fig was shared by them. There was some overlap in the dates on which the fig wasps emerged, but most females of *Wiebesia* sp. 1 had emerged before emergence of the first *Wiebesia* sp. 3 (Fig. S5).

There was consistency in the fig wasp species recorded from the five trees that were sampled in both 2009 and 2011 (Fig. S4, Fig. S5 in File S1). Most trees occupied by one species in 2009 were also colonized by the same species in 2011. While, some differences between years were also present. No *Wiebesia* sp. 1 were reared from figs on tree TH2 in 2011, though small numbers of this species were trapped in 2009, and *Wiebesia* sp. 3 was reared from figs on tree TH1, but it was not recorded on that tree’s sticky traps two years earlier. A Fisher’s exact text on these five trees confirmed that figs containing both *Wiebesia* sp. 1 and *Wiebesia* sp. 3 were present less frequently than expected if the figs were being entered randomly (n = 30 figs with just one species, 1 fig with both species present, p<0.001).

### Genetic Variation in *F. pumila*

**Figure 5:**
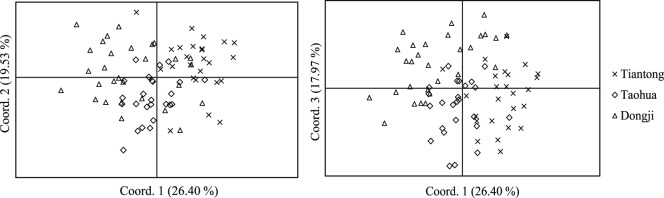
Principal coordinate analysis (PCoA) of genetic differences among 82 *F. pumila* individuals growing at Tiantong (25 trees, symbol “×”), Taohua (25 trees, symbol “◊”) and Dongji (32 trees, symbol “Δ”). The three axes together explain 63.90% of the variation.

Linkage disequilibrium was found in two loci pairs, *FP213* and *FP435*, *FP328* and *FP540*, and loci *FP328* and *FP435* were excluded from subsequent analyses. In addition, one locus (*FP327*) was found to have null alleles in all three populations, and again was not included further. We therefore analyzed population differentiation based on the remaining eight loci. The first three axes of the pairwise genetic difference-based PCoA explained a total of 63.90% of the variation (Fig. 5). An apparent cline in genetic composition was observed from Tiantong to Dongji, most likely a result of directional gene flow.

## Discussion

The flowering phenology of male *F. pumila,* with two or three synchonized annual cropping periods, strongly constrains the life cycles of the two species of pollinator fig wasp that are associated with this fig tree in Eastern China. Our study indicated that the sympatric two fig-wasp species do co-exist, but differentiate in emergence time and in micro-habitats. The two *Wiebesia* species differ in phenology, with the spring generation of *Wiebesia* sp. 1 tending to emerge from mature figs at earlier dates than do adults of *Wiebesia* sp. 3. Given their short adult life spans (few if any adult pollinators survive more than 48 hours) this difference also means that the flight periods of *Wiebesia* sp. 1 are earlier and that it enters young receptive figs before the later-emerging *Wiebesia* sp. 3. Un-pollinated figs can often remain receptive for many days [26], and if young figs of male *F. pumila* were already receptive when the first wasps emerged from the previous generation of figs then *Wiebesia* sp. 1 would be a competitive advantage, because not only can it enter figs and begin laying eggs sooner, but figs also start to become less attractive once entered by pollinators, and after a few days can cease to be suitable for entry. However, it appears that timing of production of crops of new receptive figs on each tree is influenced by the species of wasp already developing in the older figs of the previous crop on the same tree, with figs on trees that already have *Wiebesia* sp. 3 present in older figs tending to produce receptive figs of their next crop slightly later than trees where *Wiebesia* sp. 1 is already present. Synchronization of adult wasp release with production of receptive figs on the same individual has clear advantages for each male plant in summer, because it means that more fig wasps will enter their own figs, but in the spring the optimal timing of wasp release must also be highly constrained by the timing of production of receptive figs on female plants. For the wasps, the entrainment of host phenology by the wasps already present on the tree, in combination with their short adult life spans, makes it more likely that subsequent crops of figs on the same tree will be entered by the species that are already present.

### Phenologies of *Ficus pumila* and its Pollinating Wasps

North temperate dioecious fig trees display atypical and specialized flowering phenologies, characterized by population wide, relatively synchronized crops produced during set periods each year [44], [45]. This contrasts with the more general pattern found in tropical and sub-tropical fig trees, where figs are produced on different trees at different times of the year, and requires a precise match between the reproductive phenologies of the two sexes of host plants and the life cycles of their pollinating wasps. In Europe, *F. carica* has pollinators that overwinter as larvae in male figs, and when this generation of wasps emerges in the spring there are many receptive male figs available. In contrast, when the next generation of pollinators emerges in the summer, there are numerous receptive female figs and very little overlap between pollinator release and any receptive male figs [44], [46]. In Asia, *F. erecta* has the similar phenology pattern with that of *F. carica* [47]. Our study species, *F*. *pumila*, is one of the most northerly distributed *Ficus* species and has a flowering phenology that has similarities with that of *F. carica* and *F. erecta*. Although *F. pumila* and *F. erecta* are phylogenetically relatively closely related, they are not sister species with *F. carica* [48]. So, these similarities represent convergent responses to selection pressures generated by exposure to long winter periods when dispersal of their pollinators is not possible and larval development is slow or absent.

Convergence is particularly evident in the way that pollination of female figs is ensured, with the same end result obtained in two different ways. In all three species, there is one period each year when pollinators are released from male trees at a time when few receptive male figs are available, but there are many receptive female figs ready to be pollinated. Further crops are then produced on the male trees to allow populations of the pollinators to recover. In species *F. pumila* and *F. carica*, these generations of wasps are not going to have the opportunity to pollinate female figs, because the male figs in which they develop produce no pollen. This feature is not recorded for other *Ficus* species, even *F. palmata*, very closely related to *F. carica*, has developed male flowers in its overwintering figs [49]. Where the three *Ficus* species differ is in the generation of wasps that is responsible for pollinating the female figs. In *F. pumila* it is the spring (overwintering) generation of wasps that coincides with receptive female figs, whereas in *F. carica* and *F. erecta* it is adults of the second wasp generation, in the summer, that are responsible for seed set [44], [47].

The time required for figs to complete their development after they have been pollinated is highly variable between fig tree species and also strongly influenced by temperature [25], [26], [44]. The pollinators of *F. pumila* mainly have only two generations each year and development times of those individuals developing in the over-wintering male figs (up to nine months) are among the longest seen in any *Ficus* species. Given the short life span of adult fig wasps, synchrony between the emergence dates of one generation of wasps and the presence of receptive male figs is critical for the persistence of pollinator populations. The extended period of emergence of adult female wasps from each fig of *F. pumila*, which can last several days, increases the chances of overlap with receptive figs on the same trees and may also reduce interference between females as they compete to enter any figs on their natal trees. Synchrony between wasp emergence and receptive figs on female trees is equally critical for plant reproductive success. Although individual figs, if un-pollinated, can often wait for days or even weeks for pollinators to arrive [50], the reproductive success of the fig wasps, and of the plants, is usually reduced if they enter older figs [26], [51].

### Phenological Separation in Fig Wasps

The flight period of adult fig wasps was much longer at Taohua, where two pollinators were present, with *Wiebesia* sp. 1 having an earlier flight period, than at Tiantong and Dongji where there was only one (*F. pumila* population sizes are roughly similar at the three sites). The reproductive success of emerging fig wasps depends on them finding male figs to reproduce. Female figs are traps that prevent the wasps from reproducing, but it is only these wasps that can contribute to the reproductive success of the male plants where the fig wasps developed. Synchrony between D phase male figs and B phase male figs is essential for the survival of pollinator populations, while synchrony between D phase male figs and B phase female figs is necessary if their host plants are to reproduce. The extended pollinator flight period exhibited at Taohua, where both pollinator species are present, may be beneficial to female *F. pumila,* especially for those early flowering trees, if it extends the period when their figs can be pollinated. It is likely to be particularly beneficial during years when bad spring weather coincides with the flight period of one or other species of pollinator, thereby reducing their effectiveness. Conversely, a lack of synchrony between their flight period and receptive (B phase) female figs is beneficial to the wasps, as fewer will be trapped by them, but only if receptive male figs are available for them to enter. Whether the earlier flight period shown by *Wiebesia* sp. 1 puts them at an advantage, relative to *Wiebesia* sp. 3 will nonetheless mainly depend on the extent to which the flight period overlaps with B phase male figs, both on the same plant and elsewhere.

Plants growing in warmer microclimates are likely to produce figs earlier than plants in cooler situations, and early-flowering plants are clearly more favorable for *Wiebesia* sp. 1. On Taohua Island some adjacent plants consistently supported different species of fig wasp, so microclimate differences are not a likely explanation for the observed variation in spring emergence times. For example plants TH1 and TH2 were colonized by *Wiebesia* sp. 1 and *Wiebesia* sp. 3, respectively, but were growing only 60 meters apart. Genetic variation may not explain the phenological difference either, since the three populations shared the same cpDNA haplotype and belonged to the same group analyzed by the STRUCTURE based on microsatellites when including populations sampled across species distribution range (unpublished data). A possible explanation is that the two wasps occupy figs that are receptive at different time, though phenologies of *F. pumila* trees are generally synchronized. *Wiebesia* sp. 1 emerges early and enters figs at receptive stage early, while *Wiebesia* sp.3 enters those becoming receptive later.

Another possibility is that the pollinators have significant effect on the phenology of host plan. However, the extent to which host plant flowering phenology is independent of the wasps needs to be confirmed, but it appears that the timing of receptive fig production in both the spring and summer is synchronized with the release of wasps from the previous generation of figs on the same plants. Pollinator-induced changes in fig development rates have been demonstrated experimentally, with *F. citrifolia* figs containing *Pegoscapus mexicanus* shown to have longer development times than if they contain *P. franki*, which is the usual pollinator of this species [52], [53]. However, in *F. pumila* the influence of the pollinators on fig development appears to extend beyond is, to include not only the figs where wasps are present, but also younger, un-entered figs developing on the same plants. How such a mechanism could be achieved is unclear, but it has the effect that a tree containing one species of wasp is highly likely to be colonized by the same species subsequently, because the tree’s phenology has been entrained to favor the flight period of its resident pollinator. It is not known what happens when there is a break in fruiting, so that no resident fig wasps are present and the plant’s phenology is independent of resident insects.

Pollinators developing in male trees cannot influence the phenology of female trees of course, so the effects of differences in the timing of fig wasp release on the reproductive success of both the male and female plants will depend on the extent to which the release of pollinators in the spring coincides with the availability of receptive female figs. Younger female figs are typically more successful at setting good quality seed, so overlap between the start of female receptivity and pollinator release will be most favorable for the plants [26], [51]. However, in Taohua island, there are both early and late flowering females coinciding with the emergence of both pollinators. Further study should be proceeded to uncover how such phenology differentiation achieved and whether the early flowering female trees can be pollinated by both pollinators.

The earlier emergence in spring of *Wiebesia* sp. 1 adults implies an earlier or more rapid response by its over-wintering larvae to rising spring temperatures, compared with those of *Wiebesia* sp. 3. As a relatively recent colonist of eastern China, and with a more northern and westerly overall distribution than the other species [27], the response of *Wiebesia* sp. 1 to rising temperatures may reflect adaptation to the more extreme winter and summer conditions experienced in inland China. The earlier emergence of *Wiebesia* sp. 1 does not necessarily reflect a shorter overall development period from egg to adult than *Wiebesia* sp. 3, because *Wiebesia* sp. 1 also typically lays its eggs earlier.

### Potential Consequences of Differential Phenologies in Fig Wasps

The phenological difference of *F. pumila* associated to the pollinator species developing in its figs provides a mechanism for mutual competitive displacement, with trees with a resident *Wiebesia* sp. 1 population producing receptive figs at a time that is appropriate for this pollinator, and trees with a resident *Wiebesia* sp. 3 population producing receptive figs later, at a time better suited for that species. Fig wasps compete with each other in various ways. Adult females can display antagonistic responses with other females, reducing the chances they can enter a fig or the rate at which they can oviposit once inside, but such interactions have only been recorded among conspecific females and have not been observed in these *Wiebesia* species. Foundress females sharing a male fig with other females also routinely compete for oviposition sites, with an intensity varying according to how many foundresses are present. Figs also start to lose receptivity once they have been entered by a fig wasp (at a rate that can vary depending on how many foundresses have entered). Earlier arriving fig wasps can therefore make figs unavailable to fig wasps that are flying a few days later.

Competition between the two *Wiebesia* species is likely to be more intense in spring, because at that time the male plants produce fewer figs than in the summer. The different spring flight periods of *Wiebesia* sp. 1 and *Wiebesia* sp. 3 greatly reduces the likelihood of heterospecific females sharing the same fig, but there are clear opportunities for the earlier-flying *Wiebesia* sp. 1 to make figs unavailable for *Wiebesia* sp. 3, leading to asymmetric competition between the species. Long term co-existence of these species may not be possible because of this unusual form of competitive displacement, but any advantage gained by having an earlier flight periods will be reduced temporarily by the phenological entrainment of individual host trees. If the observed differences in phenology of *Wiebesia* sp. 1 and *Wiebesia* sp. 3 in coastal areas are present across mainland China, then host plant entrainment may also explain the largely mutually exclusive ranges of these species. Invasion of the range of one species of fig wasp by the other will be made difficult, because the phenologies of their host plants will often be incompatible in areas where the previous generation of figs was occupied by the other species.

Differences in adult flight periods between the two *Wiebesia* species may also have evolutionary significance. Phenological differences provide opportunities for population differentiation and speciation, especially among taxa with short adult life-spans [54]. Host race formation in *Rhagoletis pomonella*, centered around two hosts with different fruiting times, is a well known example [55]. *Wiebesia* sp. 1 and *Wiebesia* sp. 3 have only become sympatric relatively recently, as a result of an eastward expansion in range of *Wiebesia* sp. 1, and they are also not sister species [27]. This suggests that their different flight periods are unlikely to have had a role in their speciation. Their differing phenologies nonetheless have the effect of greatly reducing the chances that both species will occupy the same fig, and so reduce opportunities for hybrid fig wasp production, examples of which have not been detected between these species [27].

## Supporting Information

File S1 **This contains: Figure S1, S2, S3, S4, S5 and Table S1.** Table S1. Genotypes of individuals of *Ficus pumila* sampled in the three populations.
(DOC)
