# Supplementary material for: Competitive Exclusion among Fig Wasps Achieved via Entrainment of Host Plant Flowering Phenology
Source: PLoS One. 2014 May 21;9(5):e97783. doi: 10.1371/journal.pone.0097783 (PMC4029822; doi:10.1371/journal.pone.0097783)

**Supporting Information**

Table S1. Genotypes of individuals of *Ficus pumila* sampled in the three populations.

| population | individual | FP9 | | FP38 | | FP102 | | FP134 | | FP213 | | FP540 | | FP556 | | FP601 | |
| --- | --- | --- | --- | --- | --- | --- | --- | --- | --- | --- | --- | --- | --- | --- | --- | --- | --- |
| DJ | DJ01 | 170 | 170 | 141 | 141 | 229 | 235 | 132 | 132 | 183 | 199 | 143 | 145 | 102 | 114 | 182 | 182 |
| DJ | DJ02 | 174 | 174 | 141 | 141 | 231 | 233 | 116 | 132 | 183 | 199 | 141 | 141 | 106 | 114 | 182 | 182 |
| DJ | DJ03 | 166 | 170 | 141 | 141 | 233 | 235 | 116 | 142 | 183 | 183 | 145 | 145 | 102 | 114 | 182 | 190 |
| DJ | DJ04 | 170 | 170 | 137 | 141 | 221 | 233 | 118 | 134 | 199 | 201 | 141 | 141 | 102 | 114 | 182 | 182 |
| DJ | DJ05 | 166 | 166 | 133 | 141 | 233 | 235 | 118 | 132 | 183 | 201 | 133 | 145 | 114 | 114 | 182 | 190 |
| DJ | DJ07 | 170 | 170 | 141 | 141 | 223 | 235 | 132 | 132 | 183 | 209 | 145 | 147 | 102 | 104 | 182 | 182 |
| DJ | DJ08 | 174 | 174 | 137 | 141 | 233 | 233 | 132 | 132 | 183 | 195 | 141 | 143 | 102 | 104 | 182 | 182 |
| DJ | DJ09 | 174 | 174 | 141 | 141 | 233 | 235 | 116 | 118 | 183 | 199 | 143 | 145 | 114 | 114 | 182 | 192 |
| DJ | DJ10 | 174 | 174 | 137 | 147 | 233 | 233 | 132 | 132 | 199 | 199 | 141 | 147 | 102 | 114 | 182 | 182 |
| DJ | DJ11 | 174 | 174 | 137 | 147 | 233 | 233 | 132 | 132 | 199 | 199 | 141 | 147 | 102 | 114 | 182 | 182 |
| DJ | DJ12 | 166 | 174 | 141 | 145 | 229 | 233 | 132 | 132 | 195 | 199 | 141 | 145 | 102 | 114 | 182 | 182 |
| DJ | DJ13 | 166 | 166 | 141 | 141 | 229 | 239 | 132 | 132 | 183 | 191 | 143 | 145 | 104 | 104 | 182 | 190 |
| DJ | DJ14 | 174 | 174 | 141 | 141 | 229 | 235 | 116 | 132 | 199 | 199 | 145 | 145 | 102 | 114 | 182 | 192 |
| DJ | DJ15 | 166 | 174 | 141 | 141 | 231 | 233 | 132 | 132 | 183 | 183 | 141 | 147 | 102 | 114 | 182 | 182 |
| DJ | DJ16 | 174 | 174 | 117 | 137 | 233 | 233 | 142 | 142 | 191 | 209 | 141 | 143 | 104 | 114 | 182 | 184 |
| DJ | DJ17 | 172 | 172 | 141 | 141 | 233 | 233 | 132 | 134 | 183 | 199 | 141 | 147 | 102 | 102 | 182 | 182 |
| DJ | DJ18 | 172 | 172 | 135 | 141 | 233 | 233 | 118 | 134 | 183 | 197 | 143 | 145 | 104 | 114 | 182 | 182 |
| DJ | DJ19 | 166 | 174 | 141 | 141 | 233 | 235 | 132 | 132 | 183 | 209 | 147 | 147 | 104 | 114 | 182 | 192 |
| DJ | DJ21 | 174 | 174 | 135 | 145 | 233 | 233 | 116 | 132 | 183 | 199 | 143 | 145 | 102 | 114 | 182 | 192 |
| DJ | DJ22 | 174 | 174 | 141 | 145 | 229 | 233 | 132 | 132 | 195 | 199 | 141 | 145 | 102 | 114 | 182 | 182 |
| DJ | DJ23 | 174 | 176 | 137 | 141 | 233 | 233 | 132 | 134 | 183 | 183 | 143 | 145 | 104 | 114 | 182 | 184 |
| DJ | DJ24 | 174 | 174 | 141 | 141 | 233 | 233 | 118 | 132 | 183 | 183 | 145 | 145 | 104 | 114 | 182 | 182 |
| DJ | DJ25 | 166 | 166 | 141 | 141 | 229 | 235 | 116 | 132 | 183 | 209 | 145 | 145 | 102 | 104 | 182 | 182 |
| DJ | DJ26 | 166 | 180 | 141 | 141 | 223 | 233 | 132 | 132 | 183 | 199 | 145 | 145 | 102 | 102 | 182 | 182 |
| DJ | DJ27 | 174 | 174 | 133 | 141 | 229 | 233 | 132 | 132 | 183 | 183 | 141 | 145 | 114 | 114 | 182 | 182 |
| DJ | DJ28 | 174 | 174 | 137 | 147 | 229 | 229 | 118 | 132 | 183 | 191 | 143 | 145 | 102 | 104 | 182 | 182 |
| DJ | DJ29 | 174 | 174 | 137 | 141 | 233 | 235 | 132 | 132 | 195 | 199 | 143 | 149 | 102 | 106 | 182 | 182 |
| DJ | DJ30 | 174 | 174 | 137 | 141 | 233 | 235 | 132 | 132 | 195 | 199 | 143 | 149 | 102 | 106 | 182 | 182 |
| DJ | DJ31 | 170 | 170 | 141 | 141 | 221 | 231 | 134 | 142 | 199 | 199 | 145 | 147 | 102 | 104 | 182 | 192 |
| DJ | DJ32 | 166 | 170 | 141 | 141 | 233 | 235 | 116 | 142 | 183 | 183 | 145 | 145 | 102 | 114 | 182 | 190 |
| DJ | DJ33 | 174 | 174 | 141 | 141 | 229 | 233 | 132 | 132 | 195 | 195 | 145 | 149 | 104 | 106 | 182 | 182 |
| DJ | DJ34 | 174 | 174 | 133 | 141 | 229 | 233 | 114 | 132 | 183 | 183 | 141 | 145 | 114 | 114 | 182 | 182 |
| TH | TH02 | 166 | 174 | 137 | 141 | 235 | 241 | 132 | 132 | 183 | 195 | 145 | 145 | 100 | 100 | 182 | 190 |
| TH | TH03 | 174 | 174 | 137 | 141 | 231 | 235 | 134 | 136 | 183 | 201 | 141 | 145 | 102 | 114 | 182 | 192 |
| TH | TH04 | 174 | 176 | 137 | 141 | 233 | 235 | 134 | 134 | 183 | 195 | 145 | 145 | 102 | 114 | 182 | 182 |
| TH | TH05 | 176 | 176 | 137 | 137 | 233 | 235 | 118 | 132 | 183 | 195 | 139 | 145 | 102 | 114 | 182 | 190 |
| TH | TH06 | 166 | 176 | 135 | 141 | 229 | 229 | 118 | 132 | 183 | 195 | -9 | -9 | 102 | 104 | 182 | 190 |
| TH | TH07 | 166 | 174 | 137 | 151 | 231 | 233 | 134 | 134 | 183 | 197 | 145 | 145 | 104 | 104 | 182 | 182 |
| TH | TH08 | 166 | 170 | 137 | 141 | 233 | 233 | 132 | 142 | 183 | 197 | 145 | 145 | 104 | 104 | 182 | 190 |
| TH | TH09 | 174 | 176 | 137 | 141 | 205 | 233 | 132 | 142 | 183 | 195 | 145 | 145 | 102 | 104 | 182 | 190 |
| TH | TH10 | 166 | 174 | 137 | 141 | 205 | 233 | 132 | 132 | 183 | 195 | 145 | 145 | 102 | 114 | 182 | 182 |
| TH | TH11 | 166 | 174 | 137 | 141 | 233 | 239 | 132 | 132 | 183 | 195 | 145 | 145 | 102 | 114 | 182 | 182 |
| TH | TH12 | 166 | 174 | 137 | 141 | 233 | 233 | 132 | 132 | 183 | 195 | 141 | 145 | 102 | 114 | 182 | 182 |
| TH | TH13 | 166 | 174 | 137 | 141 | 229 | 233 | 132 | 134 | 183 | 195 | 145 | 145 | 100 | 102 | 182 | 182 |
| TH | TH14 | 166 | 174 | 137 | 141 | 221 | 229 | 132 | 132 | 183 | 195 | 143 | 145 | 102 | 104 | 182 | 182 |
| TH | TH15 | 174 | 174 | 137 | 137 | 231 | 233 | 116 | 132 | 183 | 183 | 141 | 145 | 102 | 102 | 182 | 190 |
| TH | TH16 | 166 | 174 | 137 | 141 | 233 | 233 | 116 | 132 | 183 | 195 | 141 | 145 | 100 | 100 | 182 | 190 |
| TH | TH17 | 166 | 174 | 133 | 137 | 235 | 237 | 118 | 132 | 183 | 195 | 143 | 143 | 102 | 102 | 182 | 184 |
| TH | TH18 | 166 | 174 | 137 | 141 | 229 | 235 | 118 | 132 | 183 | 195 | 141 | 145 | 100 | 100 | 182 | 184 |
| TH | TH19 | 166 | 174 | 137 | 141 | 235 | 235 | 118 | 132 | 183 | 195 | 133 | 145 | 100 | 100 | 182 | 182 |
| TH | TH20 | 174 | 176 | 137 | 137 | 223 | 233 | 132 | 134 | 183 | 199 | 133 | 145 | 102 | 102 | 182 | 182 |
| TH | TH21 | 174 | 176 | 137 | 137 | 223 | 233 | 134 | 136 | 183 | 199 | 133 | 145 | 102 | 102 | 182 | 182 |
| TH | TH22 | 174 | 176 | 137 | 145 | 233 | 205 | 118 | 134 | 183 | 183 | 145 | 145 | 102 | 114 | 182 | 182 |
| TH | TH23 | 174 | 176 | 137 | 145 | 233 | 205 | 116 | 132 | 183 | 197 | 145 | 145 | 102 | 114 | 182 | 182 |
| TH | TH24 | 166 | 176 | 137 | 137 | 205 | 235 | 132 | 132 | 183 | 197 | 141 | 145 | 104 | 114 | 182 | 182 |
| TH | TH25 | 166 | 176 | 135 | 137 | 235 | 235 | 132 | 132 | 183 | 183 | 141 | 145 | 102 | 114 | 182 | 182 |
| TH | TH26 | 166 | 174 | 137 | 141 | 231 | 235 | 118 | 130 | 183 | 183 | 141 | 145 | 100 | 100 | 182 | 182 |
| TT | TT01 | 166 | 166 | 137 | 137 | 233 | 235 | 132 | 132 | 195 | 195 | 145 | 145 | 102 | 102 | 182 | 182 |
| TT | TT02 | 166 | 176 | 141 | 141 | 205 | 233 | 132 | 132 | 183 | 195 | 141 | 141 | 102 | 102 | 182 | 182 |
| TT | TT03 | 166 | 166 | 141 | 141 | 205 | 235 | 132 | 134 | 183 | 183 | 141 | 145 | 102 | 102 | 182 | 182 |
| TT | TT04 | 166 | 176 | 137 | 137 | 223 | 223 | 132 | 132 | 183 | 199 | 141 | 145 | 102 | 114 | 182 | 182 |
| TT | TT05 | 174 | 174 | 135 | 141 | 223 | 235 | 132 | 134 | 183 | 201 | 133 | 145 | 102 | 102 | 182 | 182 |
| TT | TT06 | 166 | 176 | 137 | 141 | 233 | 235 | 132 | 132 | 183 | 183 | 133 | 145 | 102 | 114 | 182 | 182 |
| TT | TT07 | 166 | 176 | 137 | 141 | 223 | 233 | 134 | 134 | 183 | 195 | 145 | 145 | 102 | 114 | 182 | 182 |
| TT | TT08 | 166 | 176 | 135 | 141 | 235 | 235 | 134 | 134 | 183 | 195 | 145 | 145 | 102 | 102 | 182 | 182 |
| TT | TT09 | 176 | 178 | 137 | 141 | 205 | 233 | 132 | 132 | 183 | 183 | 141 | 145 | 102 | 102 | 182 | 182 |
| TT | TT10 | 174 | 176 | 137 | 141 | 233 | 235 | 132 | 132 | 183 | 201 | 141 | 145 | 102 | 102 | 182 | 182 |
| TT | TT11 | 166 | 176 | 141 | 141 | 235 | 235 | 132 | 132 | 183 | 197 | 139 | 139 | 102 | 102 | 182 | 182 |
| TT | TT12 | 166 | 176 | 137 | 145 | 233 | 235 | 132 | 132 | 183 | 195 | 139 | 141 | 102 | 102 | 182 | 184 |
| TT | TT13 | 166 | 176 | 135 | 141 | 223 | 235 | 132 | 132 | 183 | 183 | 139 | 143 | 102 | 102 | 182 | 182 |
| TT | TT14 | 166 | 176 | 137 | 141 | 235 | 235 | 132 | 142 | 183 | 183 | 145 | 145 | 102 | 114 | 182 | 182 |
| TT | TT15 | 176 | 176 | 141 | 141 | 223 | 233 | 132 | 132 | 183 | 183 | 141 | 145 | 102 | 102 | 182 | 184 |
| TT | TT16 | 166 | 174 | 141 | 141 | 223 | 233 | 132 | 132 | 183 | 183 | 143 | 145 | 102 | 102 | 182 | 184 |
| TT | TT17 | 174 | 174 | 141 | 141 | 223 | 233 | 132 | 132 | 183 | 199 | 143 | 149 | 102 | 102 | 182 | 182 |
| TT | TT18 | 166 | 166 | 141 | 141 | 223 | 235 | 132 | 132 | 183 | 201 | 143 | 149 | 102 | 114 | 182 | 184 |
| TT | TT19 | 176 | 178 | 141 | 141 | 233 | 235 | 116 | 132 | 183 | 183 | 145 | 147 | 102 | 102 | 182 | 182 |
| TT | TT20 | 166 | 166 | 135 | 141 | 233 | 235 | 132 | 132 | 183 | 201 | 143 | 143 | 102 | 102 | 182 | 182 |
| TT | TT21 | 166 | 176 | 137 | 141 | 233 | 235 | 132 | 132 | 183 | 183 | 145 | 147 | 102 | 102 | 182 | 182 |
| TT | TT22 | 166 | 176 | 141 | 141 | 235 | 235 | 132 | 132 | 183 | 195 | 133 | 145 | 102 | 102 | 182 | 182 |
| TT | TT23 | 166 | 176 | 137 | 141 | 235 | 235 | 132 | 134 | 195 | 195 | 139 | 145 | 102 | 102 | 182 | 182 |
| TT | TT24 | 176 | 176 | 137 | 141 | 233 | 235 | 132 | 132 | 183 | 183 | 141 | 145 | 102 | 102 | 182 | 182 |
| TT | TT25 | 176 | 176 | 141 | 141 | 223 | 233 | 132 | 132 | 183 | 183 | 133 | 145 | 102 | 114 | 182 | 182 |

Fig. S1. The fruiting phenologies of 14 male *F. pumila* individuals at Tiantong. A, B, C and D represent the phases of fig development, with B phase figs receptive to pollinators and D phase figs releasing pollinators. TT6 and TT11 were destroyed in August 2009. There were no spring crops on some trees (TT6 and 7).

Fig. S2. The fruiting phenologies of 10 male *F. pumila* individuals at Dongji Island. A, B, C and D represent the phases of fig development, with B phase figs receptive to pollinators and D phase figs releasing pollinators.

Fig. S3. Crop size differences between sexes and seasons in 2011 on Taohua Island. (a) Crop size variation on male trees in spring and summer; (b) Crop size variation on female trees in spring and summer.

Fig. S4. Daily captures of *Wiebesia* spp. on sticky traps placed in male *F. pumila* in 2009. Solid bars indicate *Wiebesia* sp. 1, open bars *Wiebesia* sp. 3.

Fig. S5. The identities of pollinators emerging from figs produced on eight *F. pumila* trees on Taohua Island in 2011. Each horizontal bar represents one fig. Solid bars indicate *Wiebesia* sp. 1, open bars *Wiebesia* sp. 3. Both species emerged from one fig on tree TH12.

Fig. S1


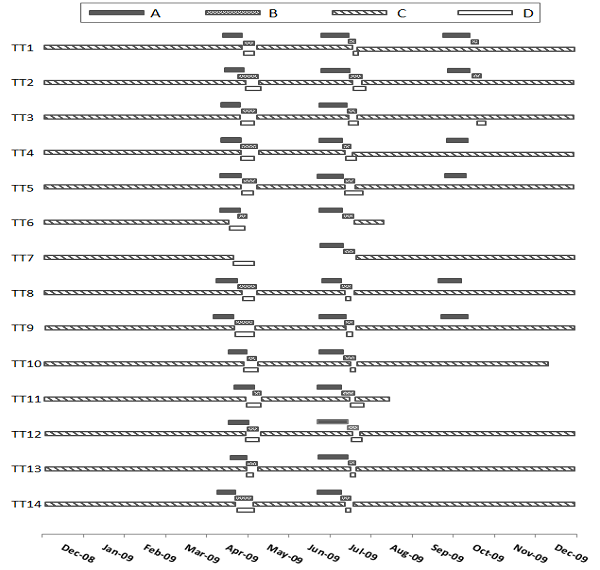


Fig. S2


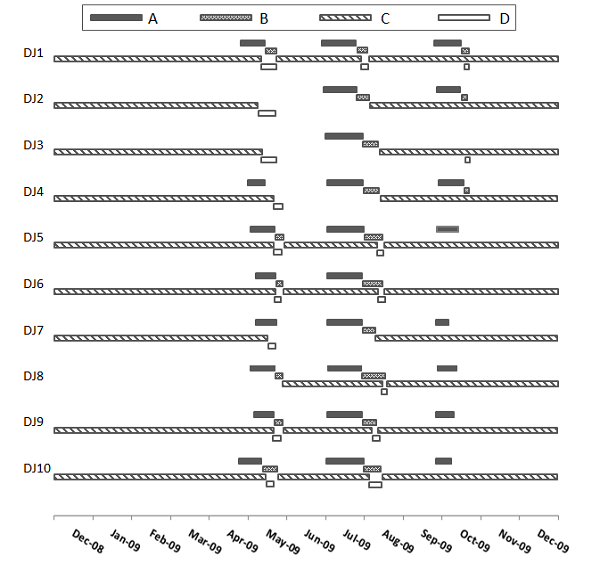


Fig. S3


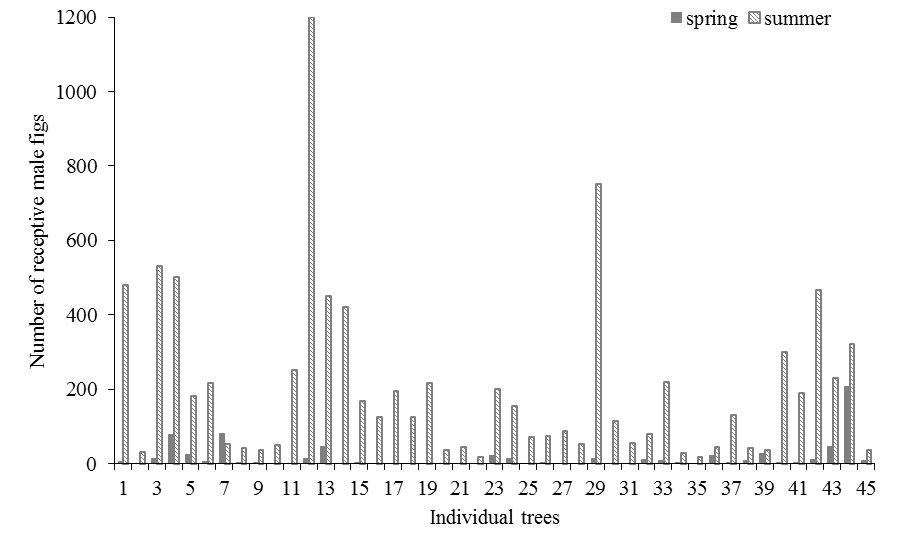


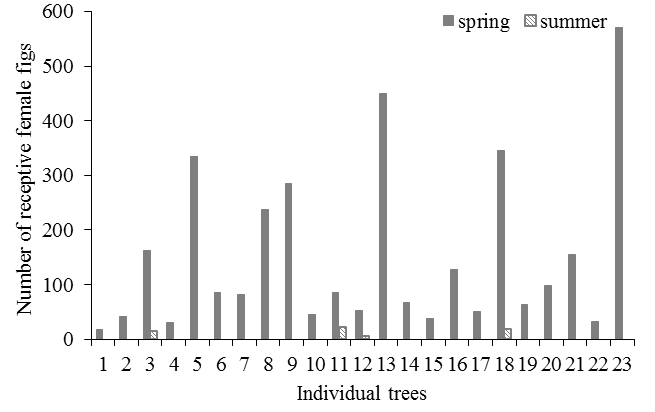


Fig. S4


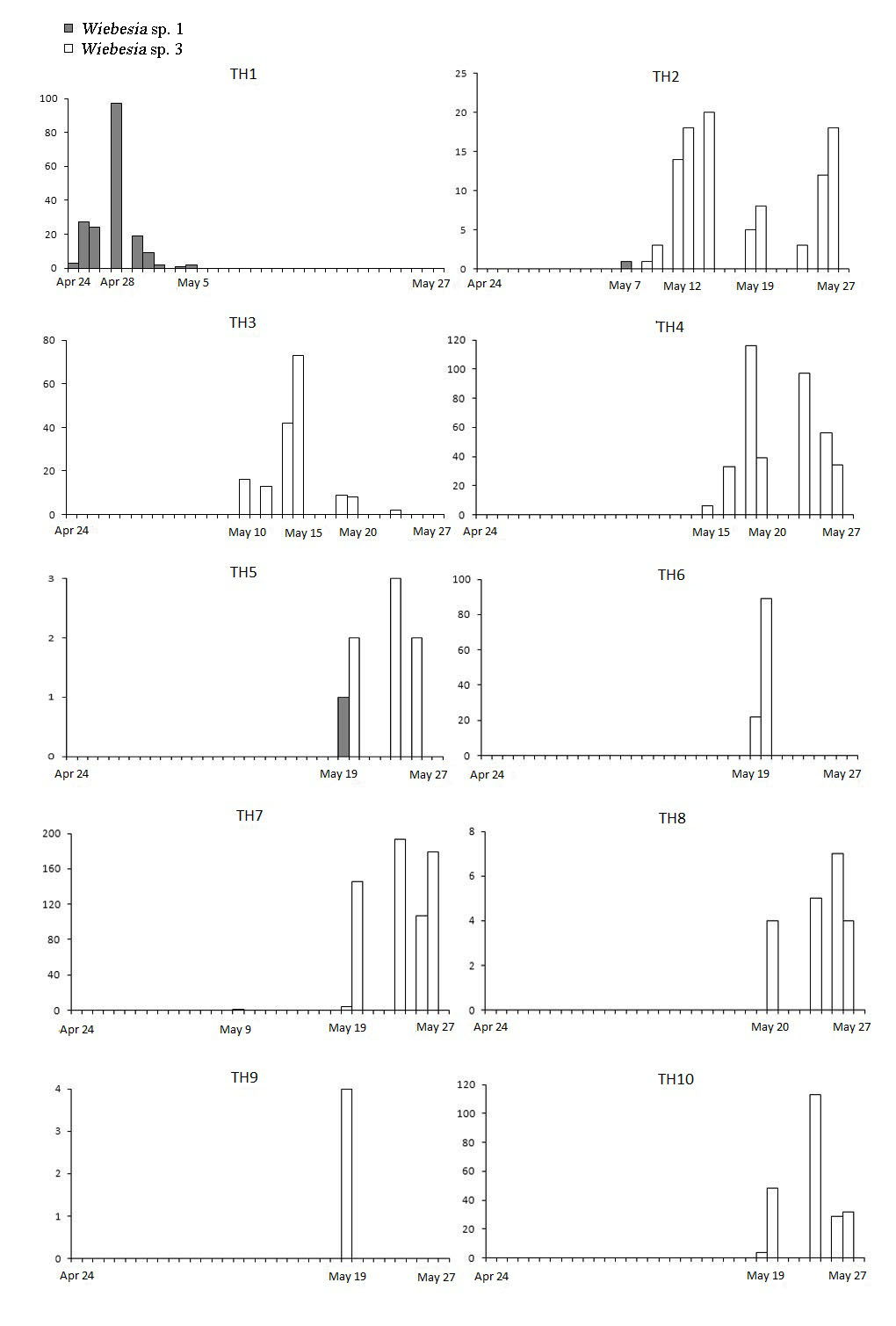


Fig. S5


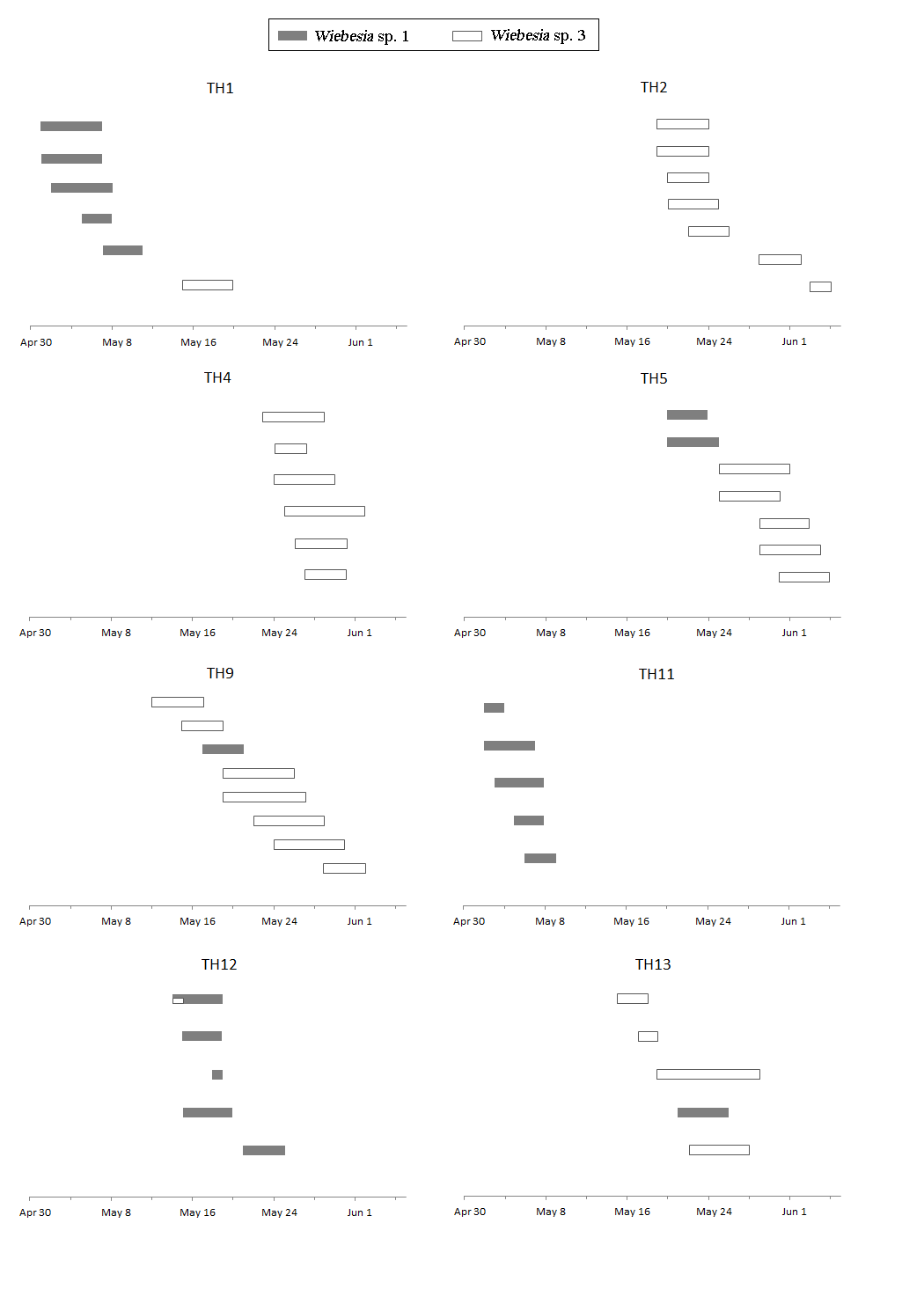

Supplement: File S1 — This contains: Figure S1, S2, S3, S4, S5 and Table S1. Table S1. Genotypes of individuals of Ficus pumila sampled in the three populations. (DOC) [file pone.0097783.s001.doc]
